# Mapping research activity on mental health disorders in Europe: study protocol for the Mapping_NCD project

**DOI:** 10.1186/s12961-016-0111-6

**Published:** 2016-05-26

**Authors:** Karen Berg Brigham, Meryl Darlington, John S. F. Wright, Grant Lewison, Panos Kanavos, Isabelle Durand-Zaleski, On behalf of the Mapping_NCD consortium, Ane Auraaen, Mursheda Begum, Reinhard Busse, Ludovica Borsoi, Oriana Ciani, Jaime Espín, Diana Gosálvez, Hala Hourani, Anshoo Lumba, María del Mar Requena, Gavin McDonough, Esther Molina‐Montes, Davina Nauth, Elena Pallari, María‐José Sánchez, Silvia Sommariva, Argo Soon, Anne Spranger, Victor Stephani, Rosanna Tarricone, Erica Visintin

**Affiliations:** URC Eco Ile-de-France (AP-HP), Hôtel Dieu, place du Parvis Notre-Dame, 75004 Paris, France; Université Paris Est Créteil Val de Marne (UPEC), 61 avenue du Général de Gaulle, 94010 Créteil, Cedex France; Department of Social Policy, London School of Economics and Political Science (LSE), Houghton Street, London, WC2A 2AE United Kingdom; Research Oncology, Bermondsey Wing, Guy’s Hospital, King’s College London (KCL), 3rd Floor, Great Maze Pond, London, SE1 9RT United Kingdom; ECEVE UMRS 1123, UEC - Hôpital Robert Debré, 48, boulevard Serurier, 75019 Paris, France

**Keywords:** Mental health, Research activities, Health priorities, Europe

## Abstract

**Background:**

Mental health disorders (MHDs) constitute a large and growing disease burden in Europe, although they typically receive less attention and research funding than other non-communicable diseases (NCDs). This study protocol describes a methodology for the mapping of MHD research in Europe as part of Mapping_NCD, a 2-year project funded by the European Commission which seeks to map European research funding and impact for five NCDs in order to identify potential gaps, overlaps, synergies and opportunities, and to develop evidence-based policies for future research.

**Methods:**

The project aims to develop a multi-focal view of the MHD research landscape across the 28 European Union Member States, plus Iceland, Norway and Switzerland, through a survey of European funding entities, analysis of research initiatives undertaken in the public, voluntary/not-for-profit and commercial sectors, and expert interviews to contextualize the gathered data. The impact of MHD research will be explored using bibliometric analyses of scientific publications, clinical guidelines and newspaper stories reporting on research initiatives. Finally, these research inputs and outputs will be considered in light of various metrics that have been proposed to inform priorities for the allocation of research funds, including burden of disease, treatment gaps and cost of illness.

**Discussion:**

Given the growing burden of MHDs, a clear and broad view of the current state of MHD research is needed to ensure that limited resources are directed to evidence-based priority areas. MHDs pose a particular challenge in mapping the research landscape due to their complex nature, high co-morbidity and varying diagnostic criteria. Undertaking such an effort across 31 countries is further challenged by differences in data collection, healthcare systems, reimbursement rates and clinical practices, as well as cultural and socioeconomic diversity. Using multiple methods to explore the spectrum of MHD research funding activity across Europe, this project aims to develop a broad, high-level perspective to inform priority setting for future research.

## Background

Mental health disorders (MHDs) encompass a broad range of conditions that together comprise a large and growing share of the burden of disease in Europe [1]. Despite the significant burden of disease, MHDs typically receive less attention and funding than other non-communicable diseases (NCDs). Governments have begun to recognize this need and are working to address the burden of MHDs, but developing an evidence-based future research agenda requires a clear picture of the current MHD research landscape, including the roles played by the broad spectrum of funding entities.

Mapping_NCD, a 2-year project funded by the European Commission, seeks to map research funding and impact in Europe for five NCDs – cardiovascular disease, respiratory disease, cancer, diabetes and MHDs – with the goal of identifying potential gaps, overlaps, synergies and opportunities, and proposing evidence-based policies to support coordinated approaches for NCD research. This study protocol describes the methodology for mapping MHD research in Europe.

Analysis of research funding and its impacts is often carried out on an institutional or national basis, where allocation may be based on a variety of criteria, such as research excellence or national disease plans, and impact analyses often rely upon bibliometric measures. Expanding the scope of such research funding analysis to encompass multiple countries and diverse funding entities requires multiple methods and entails a loss of precision because of the difficulties in delineating funding flows for research investments on such a scale. Nonetheless, estimating the extent and impact of MHD research funding is an effort worth undertaking. As Donovan noted, “*Impact is a strong weapon for making an evidence based case to governments for enhanced research support*” [2].

The Mapping_NCD project will develop a multi-focal view of the MHD research landscape by soliciting the perspectives of European funding entities, analysing the types of research undertaken in the public, voluntary/not-for-profit and commercial sectors, and contextualizing the collected data through expert interviews. In a second phase, the outputs and impact of MHD research will be explored using bibliometric analyses of scientific publications, clinical guidelines and newspaper stories reporting on research initiatives. Finally, these research inputs and outputs will be considered in light of various metrics that have been proposed to inform the allocation of research funds, including burden of disease, treatment gaps and cost of illness.

## Methods/Design

### Scope of the study

The Mapping_NCD project explores the NCD research activity across 31 European countries: the 28 European Union Member States (MSs) as well as Iceland, Norway and Switzerland. MHD research was defined as research into the causation, occurrence, presentation, diagnosis, treatments and care of disorders affecting the mental health of their sufferers in childhood, adolescence, adulthood and older age. In order to situate MHD research within the context of the relative disease burden in Europe, we will focus on ten specific MHDs for which the 2013 Global Burden of Disease (GBD) study calculated the burden as expressed in disability-adjusted life years (DALYs) [1]: addiction, alcohol use disorders, Alzheimer’s disease and other dementias, anxiety, bipolar disorder, depression, eating disorders, schizophrenia, attention deficit hyperactivity disorder and suicide/self-harm. To ensure as complete a picture of the research landscape as possible, we will compile and analyse data regarding the research activity of public, voluntary/private non-profit and commercial research funding organizations (RFOs).

The Mapping_NCD project was funded under the European Commission’s 7^th^ Framework Programme and was subject to the Commission’s ethics review process (grant agreement number 602536).

### Evidence bases for mapping research activity in Europe

**Fig. 1:**
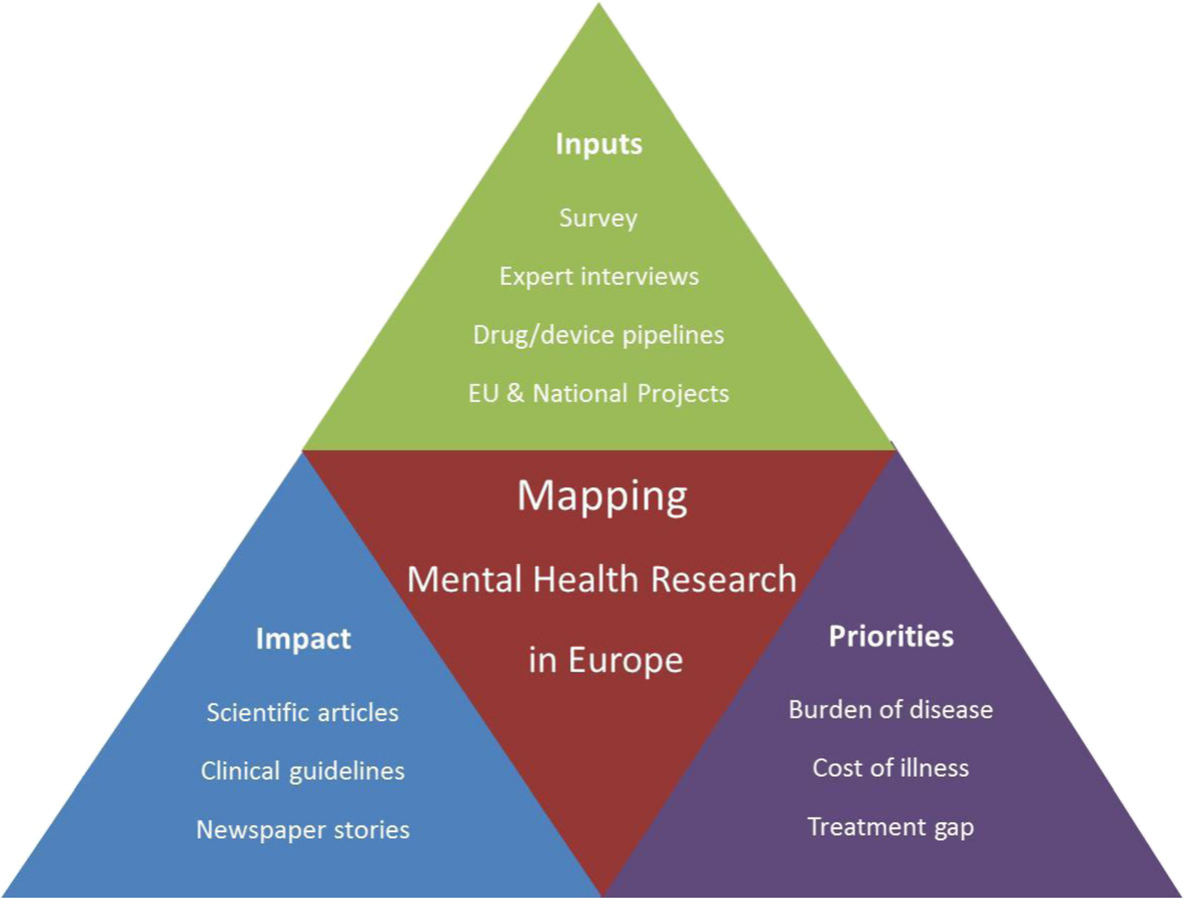
Mapping MHD research in Europe

Using an approach that combines quantitative and qualitative data, the study will develop multiple evidence bases with the goal of mapping the current scope and scale of MHD research funding in Europe. A variety of tools will be used to capture a broad-based view that encompasses the different participants and perspectives (Fig. 1).

The key element of the initial phase of the project will be an online survey of RFOs that will gather data on overall funding levels, disease focus, funding sources and priorities, types of research funded both currently and planned for the future, and expectations regarding outputs. The tool will be developed and tested in a pilot phase before being implemented across the study area. The relevant public, non-profit and private RFOs at the regional, national, supranational and EU levels will be identified through a systematic search by all consortium members across the five NCD categories. This organizational structure facilitates the identification of RFOs and the collection of survey data within countries and will enable consortium partners to overcome language barriers and ensure that data across the five disease categories are collected on a standardized basis. A baseline threshold of €0.5 million to €1 million in annual investment has been set with the goal of identifying funding that could be expected to influence the content or direction of major research programmes.

Participation in the survey will be solicited by email or Internet-based contacts. Additional website interrogations will be undertaken to gather available funding data for the identified RFOs. All non-responding RFOs will be followed-up until the close of survey, approximately 9 months after its initiation. After the data are gathered on a territorial basis, the consortium members will deliver the survey results to the members charged with the analysis of each of the five disease categories.

While the overall funding levels of RFOs can provide a macro level view of MHD research, project-based funding data provide a rich source of information regarding disease focus, types of research and project funding levels. The broad-scale view of European RFOs involved in MHD research provided by the survey will be complemented by a purposive sample of publicly-funded projects in the five largest MSs for the period 2006–2013 in order to obtain a more focussed picture of research activity in recent years. The 20 most highly-funded MHD research projects from a key public research funder in each of the five countries will be identified and analysed. Given the strong influence of the European Commission in research priority setting, MHD projects funded at the European level for the same period will be analysed. Data will be extracted from the Cordis database (http://cordis.europa.eu/home_en.html), including title, timeframe, MHD focus, type of research and level of funding. Our analysis will also include country participation, given that previous studies have found relatively low levels of participation by the newest MSs in European health-related research [3].

Commercial investment in MHD research is an important component of the overall research picture, although obtaining detailed and specific data on research and development (R&D) expenditure is challenging due to lack of transparency of information considered proprietary. We will focus our efforts on identifying the MHD products in the pharmaceutical and medical device pipelines of European and United States companies for the period 2011–2014.

To measure and compare the commitment of European and United States pharmaceutical companies to R&D investment in MHDs, including the specific disease focus, we will examine the general trends in R&D expenditure, including total R&D expenditure and as a percentage of sales or revenues. We will also explore their research pipelines in terms of MHD molecules in Phase I, II or III trials. Data for publicly-traded companies will be collected from the four most recent annual reports available on the companies’ websites (2011–2014). For privately-held companies, it is generally possible to obtain information regarding drug R&D through clinical trial databases, such as ClinicalTrials.gov. We will consider the pharmaceutical pipelines of European and United States companies at three levels: the top 20 companies in terms of R&D investment as identified by the 2014 EU Industrial R&D Investment Scoreboard (http://iri.jrc.ec.europa.eu/scoreboard14.html); companies ranked 100 to 2500 for R&D on the European Union Scoreboard; and smaller, mostly privately-held companies. This will enable us to better understand whether the disease focus and level of implication in MHD research varies depending on the size and investment capacity of the company.

To explore the medical device pipelines of the top medical device manufacturers as ranked by total revenues (http://www.mddionline.com/article/top-40-medical-device-companies), data regarding ongoing or completed trials will be obtained from ClinicalTrials.gov for the period 2011–2014. Data for medical devices with FDA pre-market approval or de novo classification will also be searched for the same period. EUDAMED, the European database of CE marked products, has existed since 2009 but is only accessible to government agencies charged with market surveillance in each country. Thus, we will search the database of the EuroScan International Network, a global network of publicly funded early awareness and alert systems for health technologies, in order to identify relevant MHD devices [4].

For both the pharmaceutical and medical device pipelines, terminated trials and those for which trial status is unknown or not verified will be excluded.

In order to contextualize the data and knowledge obtained through the preceding methods, we will undertake semi-structured interviews with key MHD experts in Europe to obtain their views on the strengths and weaknesses of the MHD research environment in Europe. We will use an interview guide with questions regarding priorities and funding trends in mental health research, the respective roles and priorities of the public and private entities, the issue of coordination and redundancy, and initiatives beyond funding that could improve MHD research in Europe.

Potential interviewees will be identified through purposive sampling with the goal of interviewing a range of experts with broad knowledge and perspectives on the MHD research landscape in Europe, including leaders of RFOs, researchers and policy experts. The recorded and transcribed interviews will be subject to qualitative analysis using the Framework Method, a highly systematic method for categorizing data with a matrix output that allows descriptive and explanatory conclusions to be organised by theme [5]. The qualitative data will then be charted into a framework matrix to allow thematic analysis across the interviews.

### Impact analyses of research

The ultimate goal of health research should be better clinical care and improved population health. The Mapping_NCD project aims to explore the pathway from research investments through to measurable impacts, and ideally, these impacts would be improvements in population health outcomes. However, the time lag from research expenditure to translation into clinical practice is estimated to be 17 years or longer [6]. Such a timeframe is beyond the scope of the present study, and thus we have selected the following proxies to measure the impact of MHD funding investments: scientific publications, clinical guidelines and newspaper stories.

One way in which research funding is considered to have had ‘impact’ is by means of knowledge production through the publication of scientific papers. Thus, we will identify the number of MHD articles and reviews through bibliometric analyses of research outputs across and within the 31 study countries for the period 2002–2013. Papers will be identified by means of a filter for which precision and recall will be tested by MHD experts, who will mark papers as relevant or not. We will analyse the publication and citation of scientific papers using the Thomson Reuters Web of Science (WoS) database and citation indexes.

The growth in research output for European MHD papers over the period will be compared to world MHD research output as well as to overall biomedical research output. The output of each country will also be explored, including the growth rate and the extent of international collaboration. These data will be further analysed by Gross Domestic Product (GDP) to determine variation in outputs based on individual countries’ economies. Finally, we will examine the research outputs by individual disorders, both across the 31 European countries and by individual country.

Citation analysis is used in allocating research funds, including at a central level to distribute funds across institutions based on aggregate statistics and at an institutional level to evaluate individual researcher and research groups for purposes of distributing funds within an institution. Citation analysis will be undertaken on the identified MHD papers using two overlapping databases, the Science Citation Index Expanded and the Social Sciences Citation Index. To measure the individual impact of research papers, 5-year actual citation impact scores will be used. Fractional counting of citations, such that a citation in a paper with n addresses would count for only (1/n)th of overall citations instead of a full point, will be used in order to facilitate comparison between different disciplinary affiliations at the paper level.

Analysis of papers referenced in clinical guidelines is an indicator of how research can inform health practice. It may also serve as an indicator of the lag time between research expenditure and potential health benefits as well as an attribution of the benefits of a particular country’s research [7]. Papers cited in MHD clinical guidelines in the 31 study countries will be identified using a macro that generates search statements to find the publications in the WoS database. The papers will then be analysed in terms of timeframe, leading country, research type, systematic reviews and mean 5-year citation score.

Finally, the results of research may be reported in news stories, which provide an additional perspective regarding the potential impact of research. While medical stories in the press target the general public, impact on health behaviours cannot be assumed and indeed may be positive or negative [8]. Media stories may also influence healthcare professionals and decision makers and in turn the policy agenda. Key newspapers in the 31 countries will be selected and their online databases and archives searched for the period 2002–2013 using simplified queries to identify stories about research involving one or more of the 10 MHDs. The related scientific publications will be identified in the WoS database. Analyses will include the focus of the research, the timeframe, the extent to which research is reported in multiple countries, particularly outside of the study country, and the journals attracting the most extensive press coverage.

### Metrics for priority setting

Various estimates of disease burden are used as metrics to identify mismatches between research investment and societal burden, including mortality, incidence, prevalence and most prominently DALYs [9]. The burden may appear very different depending on the measures used. For example, alcohol misuse has a high prevalence but a low rank in terms of years lived with disability, while schizophrenia reflects the opposite tendency, ranking third for years lived with disability but ninth for prevalence [1].

Since the first GBD study was launched in 1990, burden of disease as expressed in DALYs has been widely used as a measure of the relative magnitude of disease at the country or regional level and has informed debates about the health sector [10]. A benefit of DALYs as a metric is the fact that it integrates morbidity and mortality while also allowing them to be considered separately. Our analysis will use the 2013 DALYs for the 31 study countries from the 2013 GBD study by the Institute for Health Metrics and Evaluation [11].

While we will use MHD disease burden as measured in DALYs as a key metric in our analyses, it also has limitations, particularly as a means of allocating research funding, because it fails to account for differences in resources across countries [12, 13]. Therefore, we will also consider other measures that may help to inform research priority setting.

The treatment gap is a measure of unmet need, representing the absolute difference between the true prevalence of a disorder and the treated proportion of individuals affected by the disorder. Alternatively, the treatment gap may be expressed as the percentage of individuals who require care but do not receive treatment. The excess disability due to MHDs is partially explained by early age of onset, yet initial treatment is frequently delayed for many years. Reasons for such delays include failure to seek help because the problem is not acknowledged, a perception that treatment is not effective, a belief that the problem will go away on its own or a desire to deal with the problem without outside help. In addition, lack of knowledge about mental disorders and stigma remain major barriers to care. Other factors act as direct barriers to care, including financial considerations and issues of accessibility, as well as limited or lack of availability of certain services in some countries or for some populations. Indeed, MHDs have often been given a lower priority than physical diseases, although there are signs that this is beginning to change in Europe.

A study examined the extent of the treatment gap and found that, despite the existence of some effective and cost-effective treatments, many individuals with psychiatric disorders remain untreated [14]; 37 studies of treatment gaps were identified worldwide and revealed a high degree of unmet need for the following MHDs: schizophrenia (32%), bipolar disorder (50%), major depression (56%), generalized anxiety (58%) and alcohol use disorders (78%). We will incorporate this metric into our analyses of the data acquired for these five MHDs.

Cost of illness is another measure of the societal burden of disease. The European Brain Council (EBC) has undertaken studies of the costs of brain disorders in Europe, where 23% of years of healthy life are lost due to brain diseases at an annual total cost to the region of €386 billion. In 2005, the EBC published, for the first time, overall estimates of annual costs for brain disorders in Europe, which were updated and extended to additional disorders in 2010 [15, 16]. The EBC study was based on the best data available at the time, and modelling allowed extrapolation to countries with paucity of data, the results of which were found to be consistent with administrative data on healthcare expenditure in Europe. Cost of illness estimates, including breakdowns by country and type of cost (direct and indirect), were reported for each of the MHDs included in the Mapping_NCD study except for attention deficit hyperactivity disorder and suicide/self-harm, and we will analyse the MHD research activity and output data collected in light of the estimated cost of these disorders.

## Discussion

Since the publication of the 1990 Global Burden of Disease study, the burden of MHDs has increased worldwide [1]. From 1990 to 2013, prevalence of each of the 10 MHDs included in our study has increased, sometimes dramatically: Alzheimer’s disease and other dementias (+88%), schizophrenia (+52%) and anxiety disorders (+42%). These increases are attributable to multiple factors, including aging populations, improvements in diagnostic tools, better classification criteria and improved epidemiological studies, and underscore the need to develop improved treatments and health services.

A clearer and more comprehensive picture of the state of MHD research is essential so that limited resources may be directed to evidence-based priority areas. However, MHDs pose a particular challenge in mapping the research landscape. The complex nature and high co-morbidity of many MHDs, combined with differences in methodological standards and diagnostic instruments, make studies difficult to compare. Capturing and comparing overall disease prevalence is further challenged by variation in data collection, healthcare systems, reimbursement rates and clinical practices in European states. The significant cultural and socioeconomic differences found across Europe also make it difficult to generalize results [17]. As Knapp et al. [18] note, “*It is hard – perhaps foolishly heroic – to generalize from country to country because mental health systems, sociodemographic structures, cultural contexts, personal preferences, political priorities and economic incentives can be so very different*”.

Ideally, it would be possible to trace the beneficial effects of research funding over time. Indeed, an emerging field of study concerns estimation of the return on research investment by comparing the accrued economic benefits with its cost. A United Kingdom study estimated the returns of public and charitable research in terms of health gains and GDP gains in two therapeutic areas: cardiovascular disease and mental health [7]. Net health gains to the United Kingdom population due to MHD research, computed as the internal rate of return, were estimated at 7%, while the additional internal rate of return from GDP gains were 30%, for a total rate of return of 37%. In other words, a £1.00 investment in public/charitable MHD research produced a benefit stream equivalent to £0.37 annually in perpetuity.

One of the most significant challenges in estimating the return on research investment concerns the lag time between medical research and its impact. The United Kingdom study estimated public, charitable and private pharmaceutical research expenditure for the period 1975 to 1992 and estimated the health gains over the period 1985 to 2005. Undertaking this level of analysis across the 31 countries in our study would be extremely resource-intensive and most likely impossible at this time due to lack of necessary data. However, we raise it to encourage reflection on the need for standardization of data and the development of more sophisticated methodologies to better estimate the benefits of medical research, particularly for MHDs.

We recognize the challenges we face in our quest to map MHD research and its impact in Europe and have attempted to identify the limitations in our methodology. The maxim “*not everything that counts can be counted, and not everything that can be counted counts*” applies well to the task of measuring research and its impact. While data and resource limitations constrain the depth of our inquiry, we believe that this project will provide a high-level, multi-focal view of the current MHD research situation across Europe able to inform priorities for future research.

## Abbreviations

DALY, disability-adjusted life year; EBC, European Brain Council; GBD, Global Burden of Disease; GDP, gross domestic product; MHD, mental health disorder; MS, Member State; NCD, non-communicable disease; RFOs, research funding organizations; R&D, research and development; WoS, Web of Science
